# Whitefly Network Analysis Reveals Gene Modules Involved in Host Plant Selection, Development and Evolution

**DOI:** 10.3389/fphys.2021.656649

**Published:** 2021-04-13

**Authors:** Jiahui Tian, Haixia Zhan, Youssef Dewer, Biyun Zhang, Cheng Qu, Chen Luo, Fengqi Li, Shiyong Yang

**Affiliations:** ^1^School of Ecology and Environment, Anhui Normal University, Wuhu, China; ^2^Beijing Key Laboratory of Environment Friendly Management on Fruit Diseases and Pests in North China, Institute of Plant and Environment Protection, Beijing Academy of Agriculture and Forestry Sciences, Beijing, China; ^3^Bioassay Research Department, Central Agricultural Pesticide Laboratory, Agricultural Research Center, Dokki, Giza, Egypt; ^4^Collaborative Innovation Center of Recovery and Reconstruction of Degraded Ecosystem in Wanjiang Basin Co-Founded by Anhui Province and Ministry of Education, Anhui Normal University, Wuhu, China

**Keywords:** weighted gene co-expression network analysis, whitefly, development, host plant, co-expressed genes

## Abstract

Whiteflies are Hemipterans that typically feed on the undersides of plant leaves. They cause severe damage by direct feeding as well as transmitting plant viruses to a wide range of plants. However, it remains largely unknown which genes play a key role in development and host selection. In this study, weighted gene co-expression network analysis was applied to construct gene co-expression networks in whitefly. Nineteen gene co-expression modules were detected from 15560 expressed genes of whitefly. Combined with the transcriptome data of salivary glands and midgut, we identified three gene co-expression modules related to host plant selection. These three modules contain genes related to host-plant recognition, such as detoxification genes, chemosensory genes and some salivary gland-associated genes. Results of Gene Ontology and Kyoto Encyclopedia of Genes and Genomes analyses elucidated the following pathways involved in these modules: lysosome, metabolic and detoxification pathways. The modules related to the development contain two co-expression modules; moreover, the genes were annotated to the development of chitin-based cuticle. This analysis provides a basis for future functional analysis of genes involved in host-plant recognition.

## Introduction

The whitefly *Bemisia tabaci* (Hemiptera: Aleyrodidae) is one of the most important insect pests for major crops, especially in the sub-tropical and tropical regions around the world (Liu et al., 2007; De Barro et al., 2011). Systematic studies of the *B. tabaci* reveal convincing evidence that *B. tabaci* is a complex species including at least 35 cryptic species with extensive genetic diversity (De Barro et al., 2011; Bing et al., 2013). Among them, the Middle East-Asia Minor 1 (MEAM1) and Mediterranean 1 are considered to be the most widely distributed species worldwide, causing substantial economic damage to crops (Xu et al., 2011; Liu et al., 2012). *B. tabaci* has a wide host range with a total of 600 different plant species from different families such as: Compositae, Cruciferae, Cucurbitaceae, Euphorbiaceae, Leguminosae, Lamiaceae, Malvaceae, and Solanaceae (Mound and Halsey, 1978; Johnson et al., 1982; Elsey and Farnham, 1994; Bayhan et al., 2006; Li et al., 2011). In spite of the large-scale of host-plant use, whiteflies show diverse behavior concerning to host plant preference, oviposition, ecological adaptation as well as population size and degree of plant damage (Butler and Henneberry, 1989; Costa et al., 1991). However, the genes related to whitefly development and host-plant selection are still unknown.

One of the methods used to understand gene function and gene association from genome-wide expression is co-expression network analysis (Langfelder and Horvath, 2008; Childs et al., 2011; Liang et al., 2014). Weighted gene co-expression network analysis (WGCNA) is the most commonly used systemic biology approach used to identify the pattern of correlations among genes (Tahmasebi et al., 2019). The genes in co-expressed module, combined with functional annotations [Gene Ontology (GO) and Kyoto Encyclopedia of Genes and Genomes (KEGG)] and comparative evolution, could make the results of WGCNA more meaningful (Stuart et al., 2003; Zinkgraf et al., 2017). Thus, co-expressed modules can be helpful in understanding the function and co-expression module in genes.

In the present study, we used WGCNA analysis to construct a co-expression network among genes to identify host plant selection-related and development-related co-expression modules in the MEAM1 species of *B. tabaci*. Moreover, we accessed the function analysis and evolutionary selection pressure analysis of the key module genes.

## Materials and Methods

### Data Preparation

We downloaded the genome sequence of the *B. tabaci* MEAM1 species from www.whiteflygenomics.org (download version: MEAM1_scaffold_v1.2.fa.gz) and gene annotation GFF3 file (download version: MEAM1_v1.2. gff3.gz). The publicly available whitefly RNA-Seq transcriptome datasets deposited in the NCBI SRA database were used in the analysis (Supplementary Table 1). Data included SRA data of whitefly fed on different host plants (Malka et al., 2018) and SRA data at different developmental stages (Zeng et al., 2018). We used the alignment tool Hisat2 version 2.1.0 to map the transcriptome sequence to the MEAM1 genome, and used featureCounts version 1.6.4 to calculate the count value of the transcriptome expression of the whitefly transcriptome of different treatments, and convert the count values of each gene into TPM expression values.

### Weighted Gene Co-Expression Network Analysis

We ran the WGCNA software package on R (version 3.6.1). Before constructing the co-expression network, we filtered out samples with more than 10% missing genes, removed genes with 0 variance and genes with more than 10% missing samples. The genes were clustered into network modules using the topological overlap measure (TOM). Genes were grouped by hierarchical clustering on the basis of dissimilarity of gene connectivity (1-TOM). The co-expression clusters were produced by dynamic Mods in which the minimum size of modules was kept at 30 genes. The modules were randomly color-labeled. An adjacency matrix was built by applying a power function (β) on the Pearson correlation coefficient.

### Differential Gene Expression Analysis

We used the B-biotype whitefly midgut transcriptome (SRR835757) and salivary gland transcriptome (SRR10780450) to determine the differentially expressed genes in the midgut and salivary gland of *B. tabaci*. The calculation method of TPM expression values of genes in the midgut and salivary glands is the same as above. We used the DESeq2 software package in R (version 3.6.1; Love et al., 2014) to perform the differential expression analysis of genes (Supplementary Table 3). According to the Benjamini-Hochberg procedure, if the *p*-value is less than 0.05 after 5% FDR and the log2Fold change is greater than 2, then the gene is considered to be differentially expressed.

### Functional Annotation of Genes in Key Modules

We used the KEGG Orthology Based Annotation System (KOBAS 3.0) to perform Gene Ontology and KEGG on genes in key co-expression modules. In KOBAS, the term enrichment was defined as the Benjamini-adjusted Fisher’s exact test *p*-value. A corrected *P*-value less than or equal to 0.01 was considered statistically significant (Pu et al., 2020).

### Evolutionary Analysis of Homologous Genes in Key Modules

We choose *Bemisia* Mediterranean (MED or “Q” biotypes) species as the related group of *B. tabaci*. We employed OrthoFinder version 2.3.11 software program to calculate the orthologous genes in the B-and Q-biotypes of whitefly. KaKs_Calculator 2.0 was used to analyze the selection pressure of homologous genes in key modules.

## Results

### Gene Co-Expression Network for *B. tabaci* Was Successfully Constructed

**FIGURE 1:**
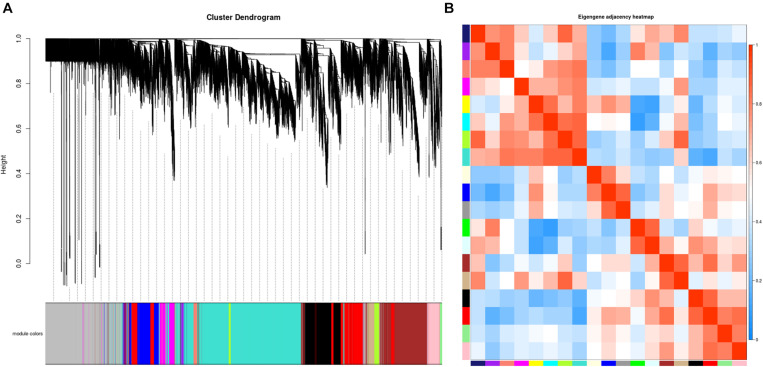
Establishment of a co-expression network in whitefly. **(A)** Gene clustering tree (dendrogram) of 15,560 genes obtained via hierarchical clustering of topological overlapping dissimilarity. **(B)** Eigengene adjacency heatmap of the 19 modules in the network. Each row and column in the heatmap corresponds to one module (labeled in color). The scale bar on the right represents the correlation strength ranging from 0 (blue) to 1 (red).

A total of 33 whitefly samples were analyzed including different developmental stages of *B. tabaci*: egg, 1–2nd instar nymphs, 3rd instar nymphs, 4th instar nymphs, males, and females. The whiteflies were fed with different host plants: sucrose-pepper, pepper, kale, eggplant, and cassava. A β value of 27 was determined to be optimal for balancing the scale-free property of the co-expression network and the sparsity of connections between genes (Supplementary Figure 1). A total of 19 co-expressed gene modules were identified after merging similar modules (Figure 1A). The network genes were then clustered and modules were detected using the dynamic tree cut method. A heatmap of eigengene correlations among the 19 modules indicated that the adjacent modules with red squares along the diagonal clustered to form several meta-modules with high correlation, suggesting that multiple modules may be involved in a similar biological process (Figure 1B).

### Identification and Function of Host Plant Selection-Related Modules in the Whitefly

**FIGURE 2:**
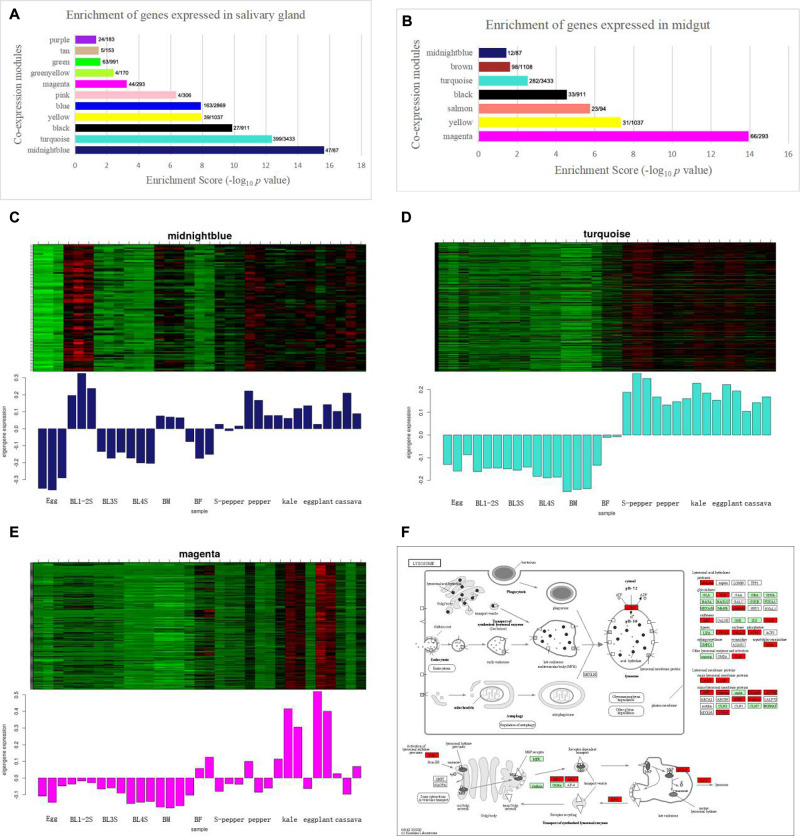
The host plant selection-related module of whitefly is performed by WGCNA. **(A)** Enrichment scores of genes expressed in salivary gland. The *x*/*y* values above the bars indicate gene numbers in the enriched category (*x*) and module (*y*). **(B)** Enrichment scores of genes expressed in midgut. The *x*/*y* values above the bars indicate gene numbers in the enriched category (*x*) and module (*y*). **(C)** The heatmap of expression pattern of genes in midnight blue module enriched in salivary gland. **(D)** The heatmap of expression pattern of genes in turquoise module enriched in salivary gland. **(E)** The heatmap of expression pattern of genes in magenta module enriched in midgut. **(F)** The KEGG enrichment of Lysosome in turquoise module with the *P*-Value of 1.5E-09.

To identify the host plant selection-related modules, we performed an enrichment analysis on the genes in the co-expression module and the differentially expressed genes in the salivary glands and midgut. There were 1168 genes differentially expressed in the salivary glands. Midnight blue module and turquoise module were enriched for whitefly genes expressed in the salivary glands (Figure 2A). There were 1151 differentially expressed genes in the midgut. Magenta module was enriched for midgut-expressed genes (Figure 2B). The heatmap and characteristic value bar graph of the expression level of the module showed that the genes in the midnight blue, turquoise and magenta modules were highly expressed in different host plants (Figures 2C–E). Cytoscape software was used to construct a visual network based on target genes, and the first 50 connectivity genes were obtained through the cytohubba plug-in. The depth of the color represents the strength of the connection. The co-expression network of the first fifty genes in midnight blue and magenta module was shown in Supplementary Figure 2. To further determine the relationship between the gene-enriched modules differentially expressed in the salivary glands, midgut and host selection, we conducted GO and KEGG enrichment analyses in these three modules. From the GO annotation (Supplementary Table 2), the midnight blue module was associated with “neuropeptide signaling pathway (GO:0007218),” followed by “neuropeptide hormone activity (GO:0005184),” and “G protein-coupled receptor signaling pathway (GO:0007186).” The turquoise module was associated with “mitochondrial translation (GO:0032543),” followed by “ATP binding (GO:0005524).” The magenta module was associated with “peptidase activity (GO:0008233),” followed by “detoxification of zinc ion (GO:0010312).” KEGG analysis of midnight blue module genes identified “Glycosphingolipid biosynthesis – globo and isoglobo series” as the most significantly enriched metabolic pathways (Supplementary Table 2). KEGG analysis of turquoise module genes identified “Lysosome” and “Starch and sucrose metabolism” as the most significantly enriched metabolic pathways (Supplementary Table 2 and Figure 2F). KEGG analysis of magenta module genes identified “Protein processing in endoplasmic reticulum” and “Metabolic pathways” as the most significantly enriched metabolic pathway (Supplementary Table 2).

### Identification and Function of Development-Related Modules in the Whitefly

**FIGURE 3:**
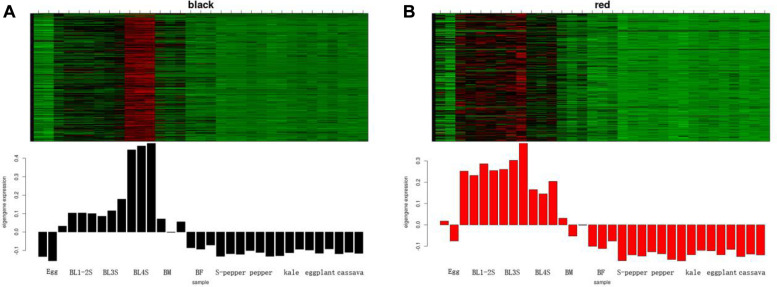
The development-related module of whitefly is performed by WGCNA. **(A)** The heatmap of expression pattern of genes in black module. **(B)** The heatmap of expression pattern of genes in red module.

According to the expression heatmap and feature value bar graph of each co-expression module, the co-expression modules related to whitefly development were determined as black and red. The genes in the black module were highly co-expressed in 4th instar nymphs (Figure 3A), and the genes in the red module were highly co-expressed in whitefly eggs and nymphs (Figure 3B). The co-expression network of the first fifty genes in the two modules was shown in Supplementary Figure 2. To determine the functions of genes in the two key co-expression modules related to development, GO and KEGG enrichment analyses were performed on the black and red genes. From GO annotation (Supplementary Table 2), the black module was associated with “chitin-based cuticle development (GO:0040003),” followed by “regulation of membrane potential (GO:0042391).” The red module was associated with “chemical synaptic transmission (GO:0007268),” followed by “G protein-coupled amine receptor activity (GO:0008227).” KEGG analysis of black module genes identified “Pentose and glucuronate interconversions” and “Ascorbate and aldarate metabolism” as the most significantly enriched metabolic pathways (Supplementary Table 2). “Neuroactive ligand-receptor interaction” was the most significantly enriched metabolic pathway in the red module.

### Evolution of Key Modules Related to Host Plant Selection and Development of the Whitefly

To study the evolutionary selection pressure of genes in co-expression modules related to host selection and development, we used the Q-biotype of whitefly as a relative group of B-biotype and performed Ka/Ks analysis on the homologous genes of the two species (Table 1). Among the modules related to host selection, 2 genes in the midnight blue module have undergone positive selection, 74 genes in the turquoise module have undergone positive selection, and 12 genes in the magenta module have undergone positive selection. Among the three host-related modules, the turquoise module had the highest percentage of genes (0.057%) undergoing positive selection. Among the development-related modules, 8 genes in the black module have undergone positive selection, and 5 genes in the red module have undergone positive selection.

**TABLE 1:** Evolutionary selection pressure of key modules related to host plant selection and development of the whitefly.

	Module	Number of co-expressed genes	Number of genes with ka/ks > 1	Max of ka/ks	Min of ka/ks	Average of ka/ks	Proportion of ka/ks > 1
Host plant-related	Midnight blue	87	2	1.2207	0.001	0.289	0.036
	Turquoise	3433	74	99	0.001	0.281	0.057
	Magenta	293	12	99	0.001	0.308	0.030
Development-related	Black	911	8	99	0.001	0.261	0.013
	Red	982	5	99	0.001	0.271	0.009

## Discussion

### WGCNA Has Been Applied for Gene Co-Expression Network Construction in Many Species

In plant research, WGCNA identified cell-type specific and endoderm differentiation-associated gene co-expression modules (Zhan et al., 2015). Pu et al. (2020) used WGCNA to identify micro-RNAs functional modules and genes of ischemic stroke. In *Myzus persicae*, WGCNA was used to identify genes with expression levels that are highly correlated in different host plants (Chen et al., 2020). Their results showed that the DE transcripts were enriched in proteolysis (including Cathepsin B). In contrast with previous research, the module related to host plant selection was also included Cathepsin B, but we also found more genes related to host plant selection, such as Odorant-binding proteins (OBPs) and chemosensory proteins (CSPs). Moreover, 19 modules were obtained by WGCNA analysis, among which midnight blue, turquoise and magenta modules were highly correlated with host plant selection. Black and red modules were highly correlated with whitefly development.

### The Differentially Expressed Genes of Saliva and the Midgut Further Validate the Modules Related to Host Plant Selection

The midgut and saliva play critical roles in mediating the interaction between herbivorous insects and their host plants (Kumar et al., 2015; Huang et al., 2020). Some proteases are highly expressed in the midgut and may reflect the ability of insects to select their appropriate host plants for development (Saikia et al., 2011). In this study, we used the EBseq package to locate genes that are differentially expressed in the salivary glands and midgut. The highly expressed genes in the salivary glands and midgut were, respectively, enriched in the identified co-expression modules. The results showed that midnight blue and turquoise were enriched in the salivary glands, and magenta was enriched in the midgut. KEGG analysis of midnight blue, turquoise and magenta showed that glycosphingolipid biosynthesis, lysosome and protein processing in the endoplasmic reticulum pathway were enriched, respectively.

Among them, lysosomal enzymes are concerned with the degradation of metabolites. Lysosomes are membrane delimited organelles serving as the cell’s main digestive compartment (Appelqvist et al., 2013) and lysosome pathway enriched 50 genes. Their functions include endocytosis, phagocytosis and autophagy. Lysosomes contain a variety of proteases and esterases that are highly expressed in the salivary glands, such as cathepsins b (CTSB). Herbivorous insects can use these cysteine proteases to break down dietary proteins, and these cysteine proteases can act as defense proteins against toxins or protease inhibitors that may be produced by host plants (Koo et al., 2008; Serbielle et al., 2009). These enzymes are very important in host selection (Huang et al., 2020; Guo et al., 2020).

### Modules Related to Host Plant Selection Contain Multiple Genes Adapted to Host Plants

The host selection module contains many detoxification related genes, such as cytochrome P450 (CYPs) and UDPGT family. CYPs is an ancient superfamily of enzymes, found in all areas of life, and involved in the metabolism of a variety of substrates. These substrates play an important role in hormone synthesis, decomposition, development and detoxification (Feyereisen, 1999; Heidel-Fischer and Vogel, 2015). In previous studies, after a transfer from eggplant to cassava, pepper and kale, the P450 and UDPGT family genes were significantly enriched in the detoxification gene family of whitefly (Malka et al., 2018). And a study on the generalist aphid species, *M. persicae*, compared colonies that were reared in parallel for 1 year on *Brassica rapa* or *Nicotiana benthamiana*, the enrichment of differentially expressed genes from the P450 and UDPGT families responding to host changes (Mathers et al., 2017). This has a striking similarity to our research.

Odorant-binding proteins and CSPs play essential roles in chemical communication and host plant selection of insects (Xu et al., 2009). The modules related to host selection identified in *B. tabaci* contain several OBPs and CSPs genes. It was reported that CSP2 in *B. tabaci* can bind the plant volatiles homoterpene (E)-3,8-dimethyl-1,4,7-non-atriene (DMNT) and its analogs, which can inhibit host selection and oviposition of whitefly (Li et al., 2020). The OBP1, OBP3, and OBP4 can recognize β-ionone, a plant volatile that can inhibit the oviposition behavior of *B. tabaci* (Li et al., 2019; Wang et al., 2019). In addition, OBP2 and OBP6 were highly expressed in the heads of *B. tabaci* adults (Wang et al., 2017; Zeng et al., 2018), and in the WGCNA analysis results, OBP2 and OBP6 were identified in the midnight blue and turquoise modules, respectively. So these two OBP genes may also be involved in the identification of host plant volatiles.

### Development-Related Modules Contain Multiple Genes Related to Chitin Formation

We identified two modules related to development of whitefly: black and red. In black and red modules, genes are highly expressed in nymphs. In the GO enrichment results, the *P*-values of chitin-based cuticle development (GO:0040003) and chitin-based embryonic cuticle biosynthetic process (GO:0008362) were 7.14E-19 and 7.17E-05, respectively. Insect growth and morphogenesis are strictly dependent on the ability to remodel chitin-containing structures (Merzendorfer and Zimoch, 2003). Chitin formation and degradation are essential for insect development. Not surprisingly, malfunction of chitin synthesis leads to developmental disorders that can be observed during embryogenesis. For example, in Drosophila, zygotic disruption of any one of the genes required for proper deposition and/or morphogenesis of the cuticle will result in embryonic mortality (Ostrowski et al., 2002). According to the results of WGCNA, we found co-expression modules related to whitefly development and determined the co-expressed genes related to development.

## Conclusion

This study aimed to identify the host plant selection-related module and development-related module in the whitefly. Midnight blue, turquoise and magenta modules were related to host plant selection based on WGCNA analysis. Black and red modules were related to whitefly development. GO and KEGG analyses highlighted “Glycosphingolipid biosynthesis,” “Lysosome,” and “Protein processing in endoplasmic reticulum pathway,” as key biological processes and metabolic pathways involved in host plant selection. The “chitin-based cuticle development” and “chitin-based embryonic cuticle biosynthetic process” were the key GO terms involved in whitefly development. This study provides a foundation for studies of the genes related to host plant selection and development in *B. tabaci*.

## Data Availability Statement

The original contributions presented in the study are included in the article/Supplementary Material; further inquiries can be directed to the corresponding author/s.

## Author Contributions

JT, SY, and FL designed the study. JT wrote the first draft of the manuscript and performed the interpretation of the results and wrote the final version of article in collaboration, which was critically revised by HZ, YD, BZ, CQ, CL, and SY. All authors approved the final version of the manuscript, and agreed to be accountable for all aspects of the work.

## Conflict of Interest

The authors declare that the research was conducted in the absence of any commercial or financial relationships that could be construed as a potential conflict of interest.
