# Supplementary figures and images for: Whitefly Network Analysis Reveals Gene Modules Involved in Host Plant Selection, Development and Evolution
Source: Front Physiol. 2021 Apr 13;12:656649. doi: 10.3389/fphys.2021.656649 (PMC8076899; doi:10.3389/fphys.2021.656649)

# Scale independence

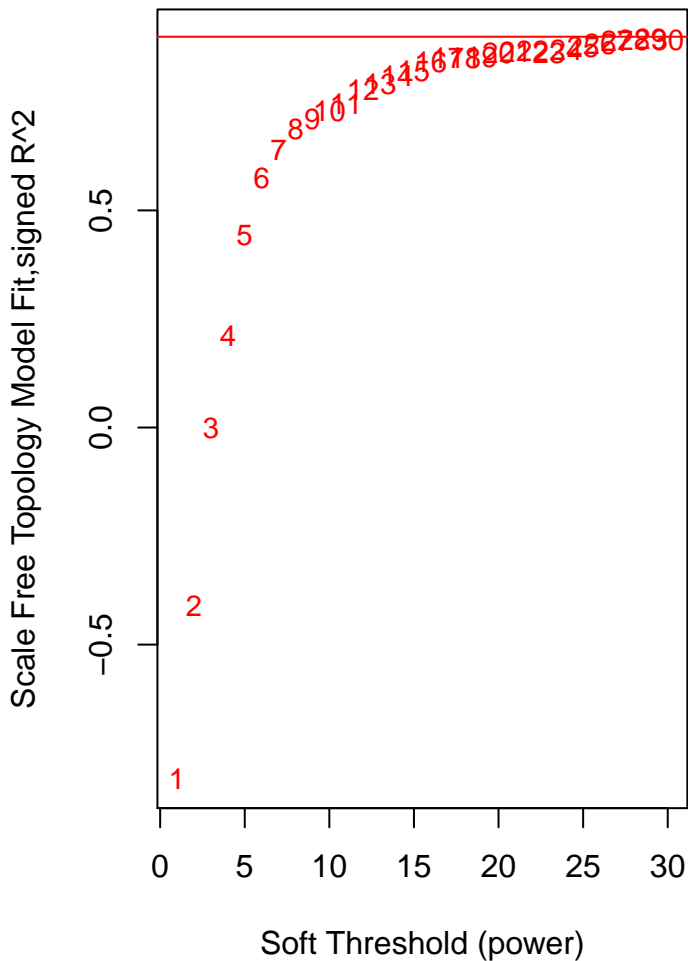

# Mean connectivity

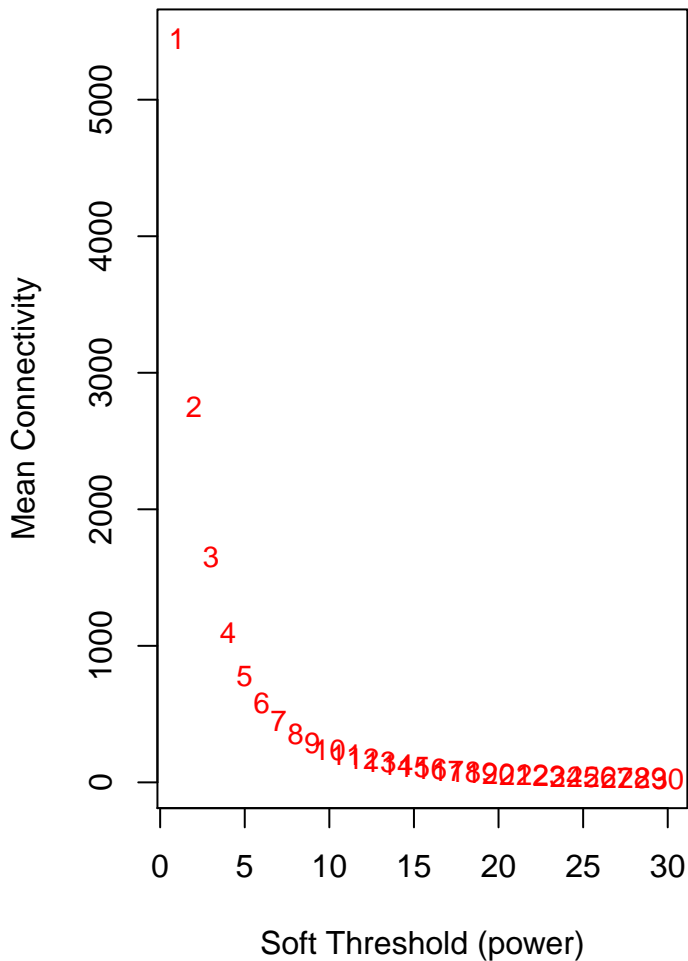

Supplement: Supplementary file 1 [file Data_Sheet_1.PDF]
