# Supplementary material for: Whitefly Network Analysis Reveals Gene Modules Involved in Host Plant Selection, Development and Evolution
Source: Front Physiol. 2021 Apr 13;12:656649. doi: 10.3389/fphys.2021.656649 (PMC8076899; doi:10.3389/fphys.2021.656649)
Supplement: Supplementary file 2 [file Data_Sheet_2.docx]

| 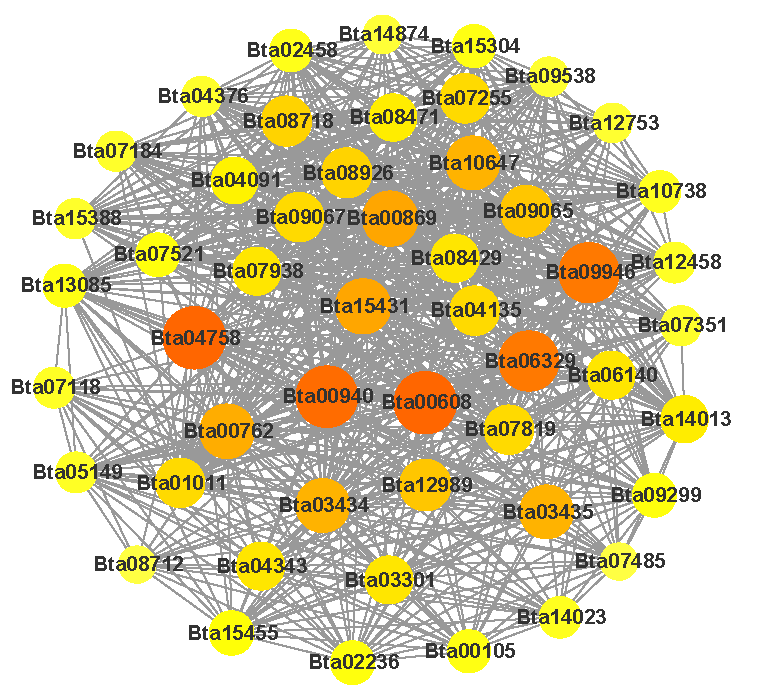  A | 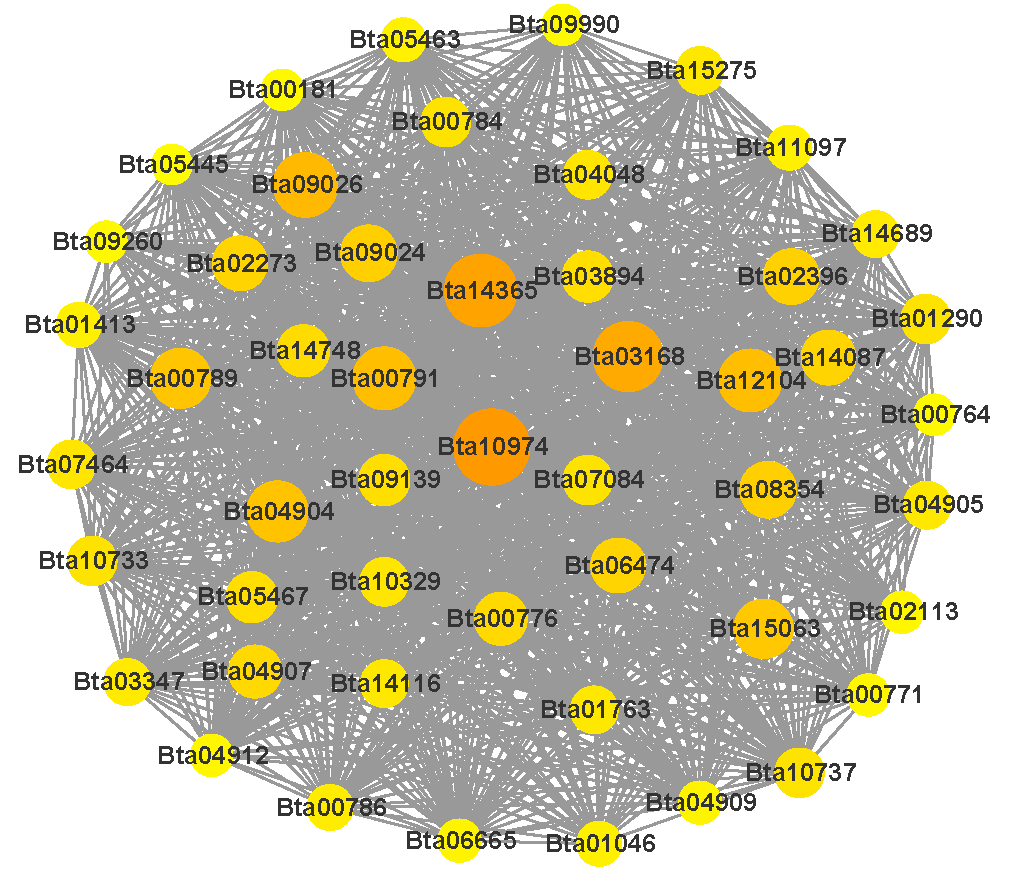  B |
| --- | --- |
| 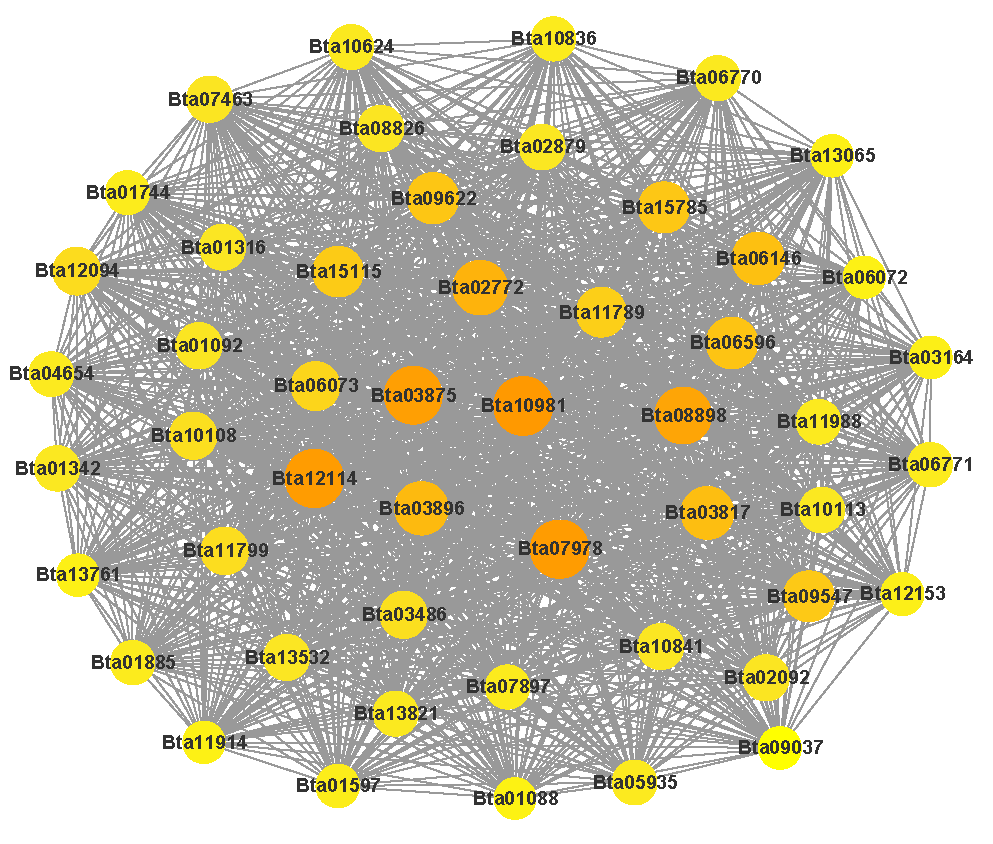  C | 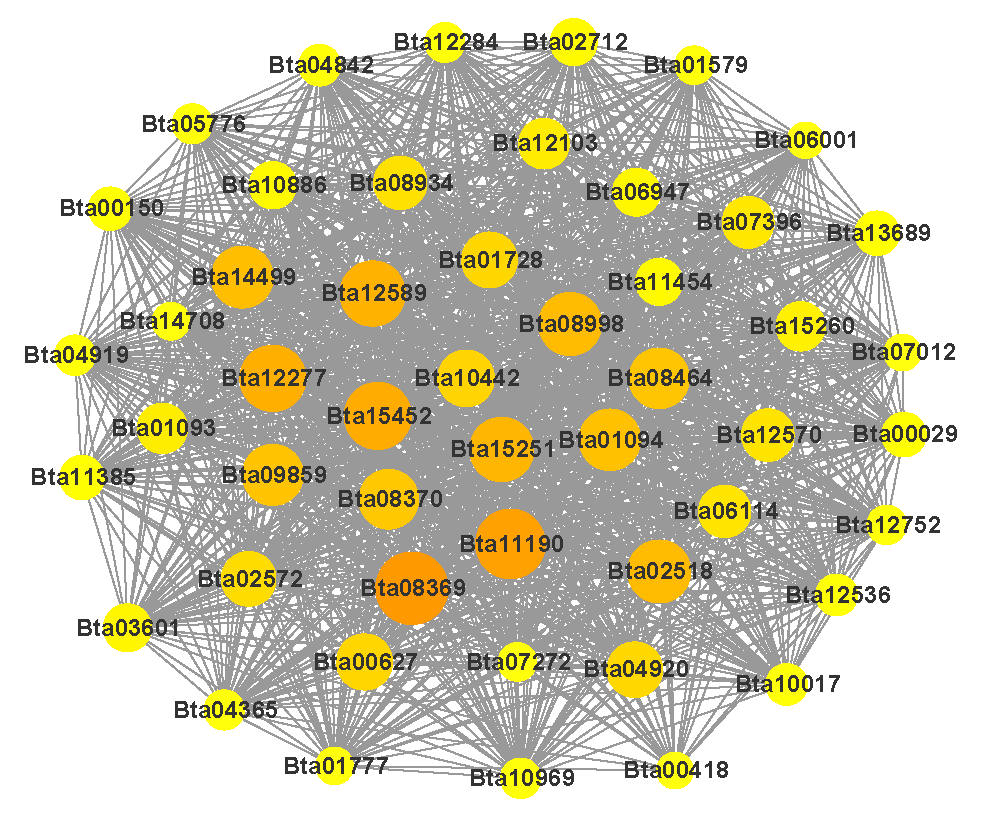  D |

Fig. S2. Co-expression network analysis of (A) midnightblue (B) magenta (C) black (D) red. Edge stood for the interaction between two genes. A degree was used for describing the importance of protein nodes (orange represented high degree and yellow represented low degree).
